# Supplementary material for: Clinical risk calculators informing the decision to admit: A methodologic evaluation and assessment of applicability
Source: PLoS One. 2022 Dec 19;17(12):e0279294. doi: 10.1371/journal.pone.0279294 (PMC9762565; doi:10.1371/journal.pone.0279294)
Supplement: S1 Table — (DOCX) [file pone.0279294.s001.docx]

| Clinical Risk Calculator Tool Name | Reference | Methods | Location |
| --- | --- | --- | --- |
| ABCD^2^ Score for TIA | Johnston et al. Lancet 2007;369(9558):283-92 | California and ABCD scores were validated and a new unified risk model was derived from their components | Derivation: 16 emergency departments in the US and 10 family practices in the UK  Validation: 16 emergency departments in the US, 9 family practices in the UK, and 1 hospital-based TIA clinic in the UK |
| ADAPT Protocol for Cardiac Risk Event | Than et al. J Am Coll Cardiol 2012;59(23):2091-8 | Prospective observational study of an accelerated diagnostic protocol | 2 hospital emergency departments in Australia and New Zealand |
| Appendicitis Inflammatory Response (AIR) Score | Andersson M, Andersson RE. World J Surg 2008;32(8):1843-9 | Prospective observational study of patients admitted with suspected appendicitis to derive and validate a risk model | 4 hospitals in Sweden  Dataset was split into derivation and validation groups |
| Clinical Index of Stable Neutropenia (CISNE) | Carmona-Bayonas A et al. Br J Cancer 2011;105(5):612-7 | Retrospective case-control study of patients with solid tumors and apparent clinical stability to compare those with complication vs those without | 1 hospital in Spain |
| CURB-65 Score for Pneumonia Severity | Lim W et al. Thorax 2003;58(5):377-382 | Retrospective analysis of data from 3 prospective CAP studies of to derive a risk model | 4 hospitals in UK, New Zealand, and the Netherlands  Dataset was split into derivation and validation groups |
| Emergency Department Assessment of Chest Pain Score (EDACS) | Than et al. Emerg Med Australas 2014;26(1):34-44 | Prospective observational study to derive and validate a risk score and create an accelerated diagnostic protocol | 2 hospital emergency departments in Australia and New Zealand  Derivation group was recruited from same hospitals over different time period as validation group |
| Emergency Heart Failure Mortality Risk Grade (EHMRG) | Lee DS et al. Ann Intern Med 2012;156(11):767-75 | Retrospective derivation and validation of risk model | 86 hospitals in Ontario, Canada  Dataset was split into derivation and validation groups |
| Glasgow-Blatchford Bleeding Score (GBS) | Blatchford O et al. Lancet 2000;356:1318-21 | Retrospective derivation of risk model and development of fast-track screen then prospective validation | Derivation: 19 emergency departments in west Scotland  Validation: 3 emergency departments in west Scotland |
| HEART Score for Major Cardiac Events | Six AJ et al. Neth Heart J 2008;16(6):191-6 | Retrospective derivation of risk model | 1 hospital-based emergency department in the Netherlands |
| History and Electrocardiogram-Only Manchester Acute Coronary Syndromes (HE-MACS) | Alghamdi A et al. Eur J Emerg Med 2019;26(5):356-361 | Secondary analyses of 3 prospective diagnostic accuracy studies to derive risk model (1 study) and validate (2 studies) | Derivation: 1 hospital in the UK  Validation: 1 hospital in the UK and data from a multicenter study (Bedside Evaluation of Sensitive Troponin study) |
| Hestia Criteria for Outpatient Pulmonary Embolism Treatment | Zondag W et al. J Thromb Haemost 2011;9(8):1500-7 | Prospective cohort study using predetermined criteria for outpatient treatment | 12 hospital-based emergency departments in the Netherlands |
| Marburg Heart Score | Bösner S et al. CMAJ 2010;182(12):1295-300 | Cross-sectional diagnostic study in primary care office with independent expert followup to derive risk model, then validation using a separate dataset | Derivation: 74 primary care practices in Germany  Validation: Data from 59 practices in Switzerland (TOPIC study) |
| MASCC Risk Index for Febrile Neutropenia | Klastersky J et al. J Clin Oncol 2002;18(16):3038-51 | Prospective observational cohort study to derive and validate risk model | International collaborative including hospitals in Australia, Canada, Europe, Pakistan, South Africa, and the US  Dataset was split into derivation and validation groups |
| Oakland Score for Safe Discharge After Lower GI Bleed | Oakland K et al. Lancet Gastroenterol Hepatol 2017;2(9):635-643 | Retrospective analysis of large dataset to derive risk model then validation using data from a separate retrospective cohort | Derivation: Large dataset of hospitals in UK (National Comparative Audit of Lower Gastrointestinal Bleeding)  Validation: 2 hospitals in the UK |
| Ottawa Heart Failure Risk Scale (OHFRS) | Stiell et al. Acad Emerg Med 2013;20(1):17-26 | Prospective observational cohort study to derive risk model | 6 emergency departments in Canada |
| Pulmonary Embolism Severity Index (PESI) | Aujesky D et al. Am J Respir Crit Care Med 2005;172(8):1041-6 | Retrospective analysis of large database for derivation of risk model, then internal and external validation | Derivation: Large database from 1 state in US (Pennsylvania Health Care Cost Containment Council)  Validation: Dataset split into derivation and validation groups plus 3 hospital-based emergency departments in Switzerland and 1 in France |
| POMPE-C Tool for Pulmonary Embolism Mortality | Kline JA et al. Thromb Res 2012;129(5):e194-9 | Preplanned secondary analysis of EMPEROR Registry dataset to derive risk model then use of data from several prospective observational cohorts for validation | Derivation: 22 emergency departments in US  Validation: Several emergency departments throughout US, New Zealand, and Europe |
| PSI/PORT Score: Pneumonia Severity Index for CAP | Fine MJ et al. N Engl J Med 1997;336(4):243-50 | Retrospective analysis of large dataset for derivation of risk model then validation in retrospective large dataset as well as prospective observational cohort study | Derivation: Large database representing 78 hospitals in 23 US states (MedisGroups Comparative Hospital Database)  Validation: Large database from 1 US state (Pennsylvania MedisGroups) and prospective observational cohort study (PORT) conducted at 5 hospitals in US and Canada |
| San Francisco Syncope Rule | Quinn JV et al. Annals of Emerg Med 2004;43(2):224-232 | Prospective cohort study to derive a risk model | 1 hospital-based emergency department in the US |
| Simplified PESI (Pulmonary Embolism Severity Index) | Jimenez et al. Arch Intern Med 2010;170(15):1383-9 | Retrospective derivation of a simplified PESI score by univariate analysis of variables from PESI, then retrospective validation of the sPESI score | Derivation: 1 hospital-based emergency department in Spain  Validation: International, multicenter registry (RIETE) |
| Troponin-Only Manchester Acute Coronary Syndromes (T-MACS) Decision Aid | Body R et al. Emerg Med J 2017;34:349-356 | Secondary analyses from total 4 prospective diagnostic cohort studies used: 1 (MACS) used to refine previous model to use troponin (instead of heart-type fatty acid binding protein) then validation using data from 3 other studies | Derivation: 1 hospital in UK  Validation: 3 hospitals in UK |
| Vancouver Chest Pain Rule | Scheuermeyer FX et al. CJEM 2014;16(2):106-119 | Prospective cohort study to derive and validate a risk model | 1 hospital-based emergency department in Canada  Derivation group was recruited from same hospital over different time period as validation group |
